# Large Language Models for Distress Rating in Korean Psycho-Oncology Interviews: Exploratory Clinician-Benchmarked Evaluation Study

**DOI:** 10.2196/91405

**Published:** 2026-09-09

**Authors:** Jaehyun Kim, Kyung-Lak Son, Chan-Woo Yeom, Won-Hyoung Kim, Sun Hyung Lee, Joon Sung Shin, Hyunsun Yang, Daehun Yoo, Bong-Jin Hahm

**Affiliations:** 1 Department of Neuropsychiatry, Seoul National University Hospital, 101, Daehak-ro, Jongno-gu, Seoul, 03080, Republic of Korea, 82 2-2072-2557; 2 Seoul Rhema Psychiatric Clinic, Gangnam-gu, Seoul, Republic of Korea; 3 Department of Psychiatry, Eulji University Uijeongbu Eulji Medical Center, Uijeongbu, Gyeonggi-do, Republic of Korea; 4 Department of Psychiatry, Inha University Hospital, Jung-gu, Incheon, Republic of Korea; 5 Genesis Lab Inc, Jung-gu, Seoul, Republic of Korea; 6 Medical Research Center, Institute of Human Behavioral Medicine, Seoul National University, Jongno-gu, Seoul, Republic of Korea

**Keywords:** large language model, psycho-oncology, mental health, distress, patients with cancer

## Abstract

**Background:**

Psychiatric distress is common among patients with cancer; yet, systematic interview-based screening remains difficult to scale in routine clinical care. Large language models (LLMs) have shown promise as scalable tools for mental health assessment, but most existing evidence is derived from clinician-authored records, translated text, or proxy data. The performance characteristics, error patterns, and explanatory behaviors of contemporary LLMs when applied to authentic, non-English psychiatric interviews remain insufficiently characterized.

**Objective:**

This exploratory study evaluated how contemporary LLMs reproduced psycho-oncologists’ item-level symptom ratings from real-world Korean psycho-oncology interviews, focusing on concordance, directional bias, and clinician-adjudicated error characteristics.

**Methods:**

Between April 2024 and May 2025, 101 adults receiving oncologic care in South Korea underwent semistructured interviews. Board-certified psycho-oncologists provided real-time, time-stamped ratings of 39 items. Generative pretrained transformer 4o (GPT-4o), Claude 3.5 Sonnet, and Gemini 2.5 Flash generated item scores and brief rationales using identical Korean zero-shot rubrics. Concordance between clinician ratings was evaluated using ordinal and binary screening metrics. A paired Wilcoxon test compared patient-level total symptom burden. Binary mismatches were clinically adjudicated as ambiguous or definite overestimation or underestimation with an 8-etiology taxonomy. Model-generated rationales were further meta-evaluated using GPT-5.4, Claude Sonnet 4.6, and Gemini Pro 3.1 across 4 dimensions: citation (use of quoted supporting statements), structure (logical organization of the rationale), mapping (consistency between the rationale and the assigned item rating), and expansion (degree of interpretive elaboration beyond the explicit transcript content). The associations between meta-evaluation results and absolute error were examined using cross-classified mixed-effects models.

**Results:**

Out of 101 participants, 88 (87.1%) were predominantly female and had breast cancer as the primary cancer type (n=70, 69.3%). Across 3931 item-level ratings, all models showed good agreement with clinicians (intraclass correlation coefficient: 0.816‐0.872), with GPT-4o showing the highest agreement. Claude 3.5 and Gemini 2.5 yielded significantly higher patient-level symptom burden (adjusted *P*<.001 and adjusted *P*=.002, respectively), whereas GPT-4o did not (adjusted *P*=.15). Clinician adjudication attributed 97 of 248 (39.1%) GPT-4o mismatches, 88 of 301 (29.2%) Claude 3.5 mismatches, and 118 of 355 (33.2%) Gemini 2.5 mismatches to intrinsic ambiguity in patient speech. Among definite errors, misapplication of severity thresholds was the predominant mechanism across models. Exploratory mixed-effects models showed that higher expansion and lower mapping scores were associated with larger absolute errors. However, the association with expansion may reflect case difficulty or ambiguity rather than causation.

**Conclusions:**

In this exploratory clinician-benchmarked evaluation of authentic interviews from a Korean psycho-oncology sample comprising predominantly women and patients with breast cancer, LLMs showed high aggregate concordance with psycho-oncologists’ item-level ratings while differing in their error profiles. These findings support further evaluation of LLM-based item-level symptom-rating approaches in psycho-oncology. Validation in larger, more diverse, and independent cohorts is needed.

## Introduction

Cancer is increasingly recognized as a “human-crisis” that profoundly disrupts psychological and emotional well-being [1,2]. Depression, anxiety, fatigue, and demoralization are highly prevalent throughout the illness trajectory and are associated with poor quality of life, reduced treatment adherence, and worse clinical outcomes [1,3]. Consequently, early detection and management of psychiatric distress are critical for comprehensive oncologic care. However, systematic screening of psychological symptoms remains challenging in daily practice because of time constraints, competing priorities, and a shortage of trained specialists [2].

A reliable assessment is indispensable; however, conventional assessment approaches are insufficient. Self-report questionnaires are constrained by comprehension and disclosure biases [4], and psychiatrist interviews, while the gold standard, are time-intensive and impractical for large-scale implementation [5]. These challenges have motivated the exploration of technological solutions to complement clinical expertise.

In recent years, large language models (LLMs) have emerged as potential adjuncts to clinical expertise [6]. With rapid advances in natural language processing, these models have demonstrated promise in medical contexts, including summarizing clinical records, extracting structured information from free-text notes, and supporting decision-making in various domains [7-9].

Their application in mental health assessment, however, remains in an early and uniquely challenging phase [10]. Unlike other areas of medicine where diagnosis and management rely primarily on explicit clinical findings, psychiatric assessment is inherently interpretive, requiring sensitivity to implicit cues, affective nuances, and contextual meanings [11]. Such nuances are embedded not only in nonverbal cues but also within verbal narratives through indirect formulation and contextual framing [12]. These subtle features introduce the risk of misclassification if AI systems are deployed without a demonstrated capacity to interpret such depth [13]. Moreover, since most pretraining data and clinical benchmarks are English-dominant and culturally Western, LLM performance may not be generalizable to languages with different pragmatic conventions and symptom-expression norms [14]. Consistent with this concern, a recent study reported higher hallucination rates and lower diagnostic accuracy when models were given Korean clinical text inputs compared to English translations, highlighting the need for language- and culture-specific validation before clinical deployment [15].

Furthermore, most LLM applications in medicine rely on clinician-authored records or other proxy texts rather than raw patient speech [9,16]. Studies based on authentic psychiatrist-patient interactions, particularly full clinical interviews, therefore, remain limited [9,17]. Addressing this gap is important, as clinical interviews remain the cornerstone of psychiatric diagnosis by directly capturing patients’ lived experiences. Although LLMs are capable of linguistic processing, their reliability in capturing the implicit aspects of psychiatric discourse remains to be validated.

Psycho-oncology is a particularly compelling context for such research. Patients with cancer face psychiatric distress shaped by existential threats, physical suffering, and complex treatment regimens [1,2]. Although the high prevalence of depression, anxiety, and fatigue in this population is well established, technological methods for systematically evaluating these symptoms have not been rigorously tested [2]. Few studies have validated these results with the judgments of psychiatrists with expertise in psycho-oncology [18,19]. The feasibility and limitations of LLM-based tools in this population remain largely unknown.

This study aimed to evaluate the concordance between contemporary LLMs and board-certified psycho-oncologists in rating symptoms from semistructured interviews with patients receiving oncologic care. The study further aimed to characterize systematic patterns of disagreement and examine how the qualitative features of model reasoning are related to rating errors. Using a dataset of routine psycho-oncology interviews with time-stamped clinician ratings across 39 symptoms and interference items, three LLMs (generative pretrained transformer [GPT]-4o, Claude 3.5, and Gemini 2.5) were evaluated using a standardized zero-shot rubric. By integrating authentic patient-psychiatrist dialogue with item-level reference ratings and a structured analysis of disagreement mechanisms, this study could address a critical gap in the evaluation of LLMs for psychiatric assessment in real-world non-English clinical settings.

## Methods

### Ethical Considerations

This study was approved by the Institutional Review Board of Seoul National University Hospital (approval number 2401-084-1501) and was conducted in accordance with the Declaration of Helsinki. Written informed consent was obtained from all participants.

### Participants

From April 2024 to May 2025, adults (≥18 y) receiving oncologic care were recruited consecutively from outpatient and inpatient oncology services at 3 secondary and tertiary hospitals in South Korea. The exclusion criteria were medical, cognitive, or linguistic limitations that precluded meaningful interviews.

### Data Collection and Interview Procedure

Interviews were conducted by 3 psycho-oncology psychiatrists using a custom application that enabled real-time symptom scoring and time-stamped tagging. During the interviews, psychiatrists assigned ratings in real time using the application, with each entry automatically time-stamped. This design created a direct link between the clinical judgment and the corresponding segment of the audio recording, producing an annotated dataset that aligned symptom scores with a conversational context.

The interviews were conducted in a semistructured format, comprising 39 evaluation items across four domains: (1) psychiatric symptoms (29 items), (2) physical symptoms (4 items), (3) psychiatric interference (3 items), and (4) physical interference (3 items). Each item was rated on a 4-point Likert scale (0‐3), with higher scores indicating greater severity or interference.

All interview recordings were transcribed using speech-to-text (STT) software, manually corrected, and time-aligned by trained assistants without a medical background. All data were fully deidentified before the analysis.

To assess clinician reference rating reliability, an independent board-certified psychiatrist, blinded to the original ratings, reevaluated 2 complementary samples: 15 high-distress cases (≥12 clinically significant items) and an additional 15 cases sampled without restriction on distress severity. Interrater agreement was high in both the high-distress sample (intraclass correlation coefficient [ICC]=0.904) and the broader-severity sample (ICC=0.899). Agreement was also high in the pooled 30-case analysis (ICC=0.911), supporting the use of these ratings as a reference standard [20].

### AI Evaluation

An LLM-based evaluation was conducted from June to August 2025 using APIs. Three LLMs were evaluated: GPT-4o (OpenAI), Claude 3.5 Sonnet (Anthropic), and Gemini 2.5 Flash (Google DeepMind), with a temperature of 0.3, a maximum token limit of 20,000, and default parameters otherwise.

All interview recordings, transcripts, clinician ratings, and adjudication data remained nonpublic throughout the study period and were not publicly released, posted online, deposited in public repositories, included in preprints, or shared as publicly accessible materials before or during the LLM evaluation. Only deidentified transcripts and item-specific evaluation inputs were used for API-based LLM inference. Therefore, contamination through publicly available training materials was considered highly unlikely.

For each patient-model combination, the complete Korean transcript and corresponding time-stamped item-specific windows were submitted in a single API call. The models were instructed to assign ordinal scores (0‐3) to each of the 39 items and generate brief textual justifications for each rating. If a call timed out or did not return a complete response conforming to the prespecified JSON schema, the same request was repeated, and the first complete schema-conforming response was retained. No additional generation was performed for that patient-model combination.

All models were guided by an identical zero-shot prompt in Korean that combined explicit evaluation rubrics with schema-constrained outputs and reasoning justifications. The prompt assigned models the role of a psychiatrist specializing in psycho-oncology. The instructions specified a fixed JSON structure with predefined keys for all items and required ratings on a standardized 4-point rubric anchored to symptom frequency, duration, and functional impairment over the preceding 1 to 2 weeks. The distinction between scores of 0 to 1 (subclinical) and 2 to 3 (clinically significant) was emphasized as the threshold for clinical significance. Conservative scoring was operationalized within the prompt by requiring clear transcript evidence for clinically significant ratings and by instructing models to assign lower scores when evidence regarding symptom frequency, duration, or functional impact was unclear or insufficient. The prompt also included interpretation guidelines to account for potential STT errors, such as phonetic misrecognitions, fragmented speech, and indirect emotional expressions.

During prompt development, we evaluated 2 rubric formats. One format used a single standardized 4-point rubric applied uniformly across all symptom items, whereas the other provided symptom-specific descriptions for scores 0, 1, 2, and 3 for each item. The standardized rubric format was selected for the final evaluation because pilot review showed more consistent application of shared scoring criteria across items, including symptom frequency, duration, and functional impact. We then compared 2 input-context formats. In the first, the model received only the transcript excerpt corresponding to each tagged symptom. In the second, the model received the complete interview transcript together with the item-specific excerpt (ie, the portion of the interview where the symptom was mentioned). The latter format was selected to preserve the broader clinical context of the interview.

These development steps concerned the general rubric and contextual input structure rather than model-specific prompt tuning. The final prompt was fixed before the main evaluation and applied identically across all models. No model-specific prompt modifications or few-shot demonstrations containing example transcripts, completed item ratings, or model-output examples were used. The only examples included in the prompt were generic anchor expressions embedded in the scoring rubric. An English translation of the full prompt is provided in Multimedia Appendix 1.

### Clinician-Adjudicated Error Analysis

Binary mismatches (clinically significant symptoms defined as score ≥2) were manually reviewed and classified using a taxonomy developed and refined through consensus within a multidisciplinary team that included clinical experts and data scientists. Selected cases were reviewed in multidisciplinary team meetings for calibration of the operational definitions and consistency of taxonomy application. A single psychiatrist served as the primary adjudicator and performed the case-level classification with explicit model-source labels masked. For each mismatch, the adjudicator reviewed the target item, the deidentified transcript and item-specific segment, the clinician and model ratings, and the model-generated rationale.

Disagreements were categorized as ambiguous (insufficient or equivocal transcript evidence) or definite overestimation or underestimation, with directional errors further classified into 8 etiologies (Table 1). Reproducibility was assessed through an independent, model-blinded review of 150 mismatches stratified by model and initial adjudication category, yielding high interrater agreement for the ambiguous-versus-definite classification (Cohen κ=0.82). To provide a clearer understanding of ambiguous disagreements, 3 deidentified and translated examples are presented in Multimedia Appendix 2.

**Table 1.** Clinician-adjudicated error taxonomy for large language model-clinician discrepancies.

Subtype	Definition
Implicit cues	Misinterpretation of subtle or indirect emotional cues, either by overattributing or underrecognizing clinically meaningful affective signals.
Other information	Use of information drawn from the wrong symptom domain, reflecting misalignment between the evaluated item and the evidence selected from the transcript.
Comprehension	Errors arising from misunderstanding, mis-parsing, or insufficiently attending to the literal content of the patient’s statements.
Criteria application	Incorrect application of standardized scoring criteria, including misjudgment of symptom frequency, duration, or functional impact.
Personality attribution	Confusion between stable personality characteristics and acute clinical symptoms, resulting in distorted severity assessment.
Somatic confounding	Misattribution between physical and psychiatric symptoms, leading to overestimation or underestimation of psychological distress.
Temporal misalignment	Incorrect interpretation of the temporal reference window, including misclassification of symptom timing relative to the 1- to 2-week evaluation frame.
Construct misclassification	Incorrect mapping of a symptom expression to the wrong clinical construct, reflecting confusion in symptom definition rather than mis-selection of transcript evidence.

### Automated Meta-Evaluation of Reasoning

To systematically investigate the qualitative characteristics of the LLM outputs, an automated meta-evaluation framework was applied to all model-generated responses between June and July 2026. To reduce dependence on any single evaluator model family, model-generated rationales were independently meta-evaluated by GPT-5.4, Claude Sonnet 4.6, and Gemini Pro 3.1 using the same rubric, with explicit source-model identifiers removed. Reasoning outputs were assessed across 4 dimensions: citation (specificity of referenced evidence), structure (evidence, interpretation, and score completeness), mapping (rules and consistency between interpretation and score), and expansion (extrapolation beyond the transcript). For each rationale and dimension, the arithmetic mean of the 3 independently assigned scores was used as the meta-evaluation score. In a 10-patient subset, agreement between the mean meta-evaluation scores and psychiatrist ratings ranged from ICC (A,1)=0.646 to 0.822 across dimensions. Detailed reliability methods and results are provided in Multimedia Appendix 1.

The meta-evaluation prompt was developed independently of the primary symptom-rating prompt. During prompt development, the rubric was refined through pilot review to improve interpretability and reduce common scoring artifacts, including over-rewarding verbose rationales, penalizing appropriate documentation of symptom absence, or relying on keyword-based scoring. After pilot review, the prompt was finalized and applied uniformly to the full set of model-generated rationales. The parameters were a temperature of 0.15 and a maximum token count of 131,702. An English translation of the full prompt is provided in Multimedia Appendix 1.

### Statistical Analysis

Statistical analyses were performed using Python (version 3.12; libraries: NumPy, SciPy, pandas, scikit-learn, and statsmodels). All hypothesis tests were 2-sided, with the threshold for statistical significance set at *P*<.05. To address the multiplicity of related tests (patient-level directional bias, meta-feature comparisons, and mixed-effects models), *P* values were adjusted using the Benjamini-Hochberg false discovery rate. For 2-df Wald tests comparing source-model-specific ICCs across the 4 meta-evaluation dimensions and the post hoc ambiguity analyses, the Holm procedure was applied.

Because this study was designed to estimate model agreement, directional bias, and error patterns rather than to test a single confirmatory hypothesis, all consecutively recruited eligible participants during the study period were analyzed. Although each interview contributed up to 39 item-level evaluations, these observations were clustered within patients and were not treated as independent patient-level cases. To assess the stability of key performance estimates, we calculated 95% CIs using nonparametric patient-level cluster bootstrapping with 2000 resamples. In each bootstrap resample, patients rather than individual item-level observations were sampled with replacement, and all item-level observations from each selected patient were retained.

Descriptive statistics were used to summarize the participant demographics.

The concordance between LLM outputs and psychiatrist ratings on a 0 to 3 ordinal scale was assessed using quadratic weighted kappa (QWK) and ICC (2,1). The exact accuracy was also reported. For screening performance, scores were dichotomized (0‐1 vs ≥2) to compute sensitivity, specificity, positive predictive value (PPV), negative predictive value (NPV), and *F*_1_-score. As a sensitivity analysis, the 170 patient-item pairs classified as ambiguous for any of the 3 models were excluded identically from all models, and the binary screening metrics were recalculated. The patient-level directional bias was evaluated by comparing the total clinically significant symptom counts per patient using the paired Wilcoxon signed-rank test.

Post hoc exploratory analyses of ambiguous mismatches were performed to examine whether the proportion of adjudicated mismatches classified as ambiguous differed across models or clinical domains. A total of 904 binary mismatches were analyzed using patient-clustered logistic generalized estimating equation models, which accounted for multiple mismatch observations from the same patient. Ambiguity status (ambiguous vs definite error) was the outcome, and model and clinical domain (psychiatric symptoms, physical symptoms, psychiatric interference, and physical interference) were included simultaneously as predictors. Following the global model test, pairwise model contrasts were estimated. To examine whether model differences varied across clinical domains, we tested the model-by-domain interaction. To examine whether model differences varied according to patient characteristics, separate domain-adjusted models tested interactions between model and standardized age, standardized clinician-rated symptom burden, major depressive episode status, and sex.

To examine whether reasoning styles differed across the models, the distributions of the meta-evaluation scores were compared using the Friedman test. To evaluate associations between rationale characteristics and prediction error while accounting for cross-classified data structures, mixed-effects models were fitted with the absolute error as a dependent variable. Meta-evaluation scores were included as fixed effects and were *z*-standardized within each model. Clinical domain (psychiatric symptoms, physical symptoms, psychiatric interference, and physical interference) was included as a 4-level fixed effect to account for systematic differences in error across domains. Random intercepts were specified for patients and items to account for within-patient clustering and item-level heterogeneity, respectively. The models were estimated using restricted maximum likelihood. As a sensitivity analysis, the same model specification was applied to the 10-patient subset using the human reference meta-evaluation scores. The resulting coefficients were compared with those obtained using the mean scores assigned by the 3 evaluator models in the same 10-patient subset and in the full cohort.

### Reporting Guideline

The TRIPOD-LLM (Transparent Reporting of a Multivariable Prediction Model for Individual Prognosis or Diagnosis–Large Language Model) reporting guideline was used to guide the reporting of this study. The completed checklist is provided in Checklist 1 [21].

## Results

### Participant Characteristics

This study included 101 patients receiving oncology treatment or follow-up care. Each interview yielded up to 39 symptom-level evaluations. Eight symptom tags were excluded because of incomplete or corrupted audio recordings that precluded reliable transcription. The final dataset comprised 3931 symptom evaluations. Demographic characteristics of participants are shown in Table 2. The mean age of participants was 52.8 (SD 9.2) years, and the majority (n=88, 87.1%) were female participants. The most common primary cancer was breast cancer (n=70, 69.3%). Most participants (n=85, 84.2%) were receiving treatment after initial diagnosis or recurrence, while a smaller proportion were in posttreatment follow-up, remission follow-up, palliative care, or had unknown treatment status. Major depressive episodes were identified in 17.8% (n=18) of the participants. The distribution of clinician-rated symptom scores was skewed toward lower severity.

**Table 2.** Demographic characteristics of participants.

Demographic characteristics	Values, n (%)
Sex	
Male	13 (12.9)
Female	88 (87.1)
Age (y)	
21-30	1 (1.0)
31-40	9 (8.9)
41-50	36 (35.6)
51-60	33 (32.7)
61-70	19 (18.8)
71-80	3 (3.0)
Cancer type	
Breast	70 (69.3)
Colorectal	9 (8.9)
Lung	5 (5.0)
Stomach	4 (4.0)
Pancreatic	3 (3.0)
Ovarian	2 (2.0)
Others	8 (8.0)
Treatment status	
Initial treatment	69 (68.3)
Posttreatment surveillance	9 (8.9)
Treatment for recurrence	16 (15.8)
Remission follow-up	1 (1.0)
Palliative care	2 (2.0)
Unknown	4 (4.0)
MDE^a^	
Mild	3 (3.0)
Moderate	13 (12.9)
Severe	2 (2.0)
Total	18 (17.8)
Symptom score	
0	2106 (53.6)
1	1082 (27.5)
2	657 (16.7)
3	86 (2.2)

a MDE: major depressive episode.

### Primary Diagnostic Performance

Across all 3 models, the concordance with board-certified psychiatrists on the 0 to 3 ordinal scale was high (Table 3 and Figure S1 in Multimedia Appendix 3). GPT-4o showed the highest agreement (ICC=0.872), followed by Claude 3.5 (ICC=0.838) and Gemini 2.5 (ICC=0.816).

**Figure 1.**
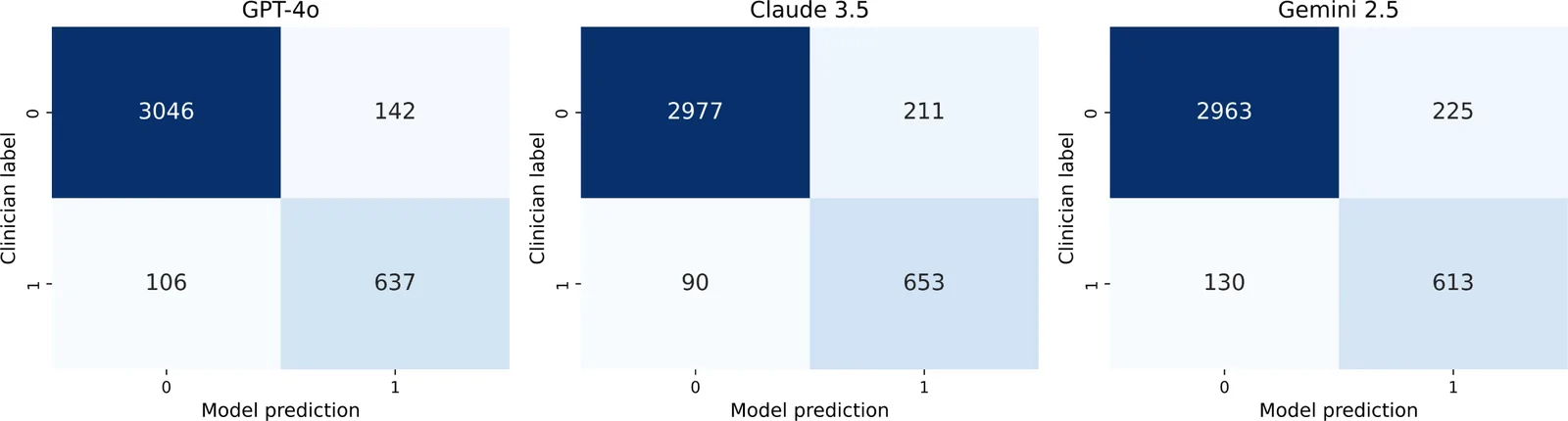
Binary confusion matrices for GPT-4o, Claude 3.5, and Gemini 2.5 Binary confusion matrices compare each model’s dichotomized predictions (0: score <2, clinically absent or subclinical; 1: score ≥2, clinically significant) with clinician ratings across 3931 symptom evaluations. The cells indicate true negatives, false positives, false negatives, and true positives for each model.

When scores were dichotomized at ≥2 to indicate clinically significant items, all models demonstrated high performance (Table 3 and Figure 1). GPT-4o showed the highest *F*_1_-score (0.837), followed by Claude 3.5 (0.813) and Gemini 2.5 (0.775). In the sensitivity analysis excluding ambiguous mismatches, *F*_1_-scores were numerically higher for all 3 models, while the model ordering remained unchanged (Table S1 in Multimedia Appendix 3).

**Table 3.** Symptom rating performance of large language models, including item-level exact agreement (ordinal accuracy), ordinal reliability metrics, and binary screening performance^a^.

Evaluation measure	GPT-4o (95% CI)	Claude 3.5 (95% CI)	Gemini 2.5 (95% CI)
Ordinal			
Accuracy	0.843 (0.823‐0.862)	0.797 (0.770‐0.824)	0.794 (0.769‐0.819)
ICC^b^	0.872 (0.857‐0.886)	0.838 (0.818‐0.853)	0.816 (0.792‐0.837)
QWK^c^	0.872 (0.857‐0.886)	0.838 (0.818‐0.853)	0.816 (0.792‐0.837)
Binary			
Sensitivity	0.857 (0.821‐0.890)	0.879 (0.844‐0.909)	0.825 (0.782‐0.861)
Specificity	0.955 (0.943‐0.966)	0.934 (0.917‐0.949)	0.929 (0.912‐0.943)
PPV^d^	0.818 (0.771‐0.856)	0.756 (0.699‐0.804)	0.732 (0.666‐0.784)
NPV^e^	0.966 (0.958‐0.974)	0.971 (0.962‐0.978)	0.958 (0.945‐0.969)
*F*_1_-score	0.837 (0.805‐0.864)	0.813 (0.775‐0.844)	0.775 (0.731‐0.813)
Adjusted *P* value	.15	<.001	.002

a Values are point estimates with patient-cluster bootstrap. Ordinal metrics were calculated on the original 4-point scale (0‐3). Binary metrics were computed using a clinical threshold of ≥2. Intraclass correlation coefficient (2,1) and quadratic weighted kappa were calculated separately; their identical values to 3 decimal places reflect rounding. Adjusted *P* values indicate Benjamini-Hochberg false discovery rate adjusted *P* values from paired comparisons of patient-level total symptom burden (sum of binary flags) between each model and clinician ratings.

b ICC: intraclass correlation coefficient.

c QWK: quadratic weighted kappa.

d PPV: positive predictive value.

e NPV: negative predictive value.

### Systematic Directional Bias in Symptom Ratings

Directional error patterns for binary classifications (score ≥2 vs <2) are summarized in Figure 1. Across all models, overestimation errors (LLMs: ≥2, clinicians: <2) were more frequent than underestimation errors (LLMs: <2, clinicians: ≥2), but the relative proportions differed by model. Overestimation accounted for 142 of 248 (57.3%) errors for GPT-4o, 211 of 301 (70.1%) errors for Claude 3.5, and 225 of 355 (63.4%) errors for Gemini 2.5 (Figure 1).

Paired Wilcoxon signed-rank tests comparing the total symptom burden between each LLM and the clinician reference standard showed no statistically significant difference for GPT-4o (adjusted *P*=.15), whereas Claude 3.5 (adjusted *P*<.001) and Gemini 2.5 (adjusted *P*=.002) yielded higher total burdens than the clinicians (Table 3).

### Clinician-Adjudicated Error Taxonomy

All binary mismatches between LLM and clinician ratings were classified by the primary adjudicator using the consensus-developed error taxonomy (Table 1), and the resulting classification frequencies are summarized in Table 4*.* Ambiguous cases accounted for a substantial proportion of disagreements across all models, comprising 88 of 301 (29.2%) mismatches for Claude 3.5, 118 of 355 (33.2%) mismatches for Gemini 2.5, and 97 of 248 (39.1%) mismatches for GPT-4o. A global difference across models was detected (Wald *χ*²_₂_=6.58; *P*=.04). In the Holm-adjusted pairwise comparisons, GPT-4o had higher odds of ambiguity than Claude 3.5 (adjusted *P*=.03), whereas the other model contrasts were not significant (Table S3 in Multimedia Appendix 3). Neither the clinical-domain effect nor the model-by-domain interaction was statistically significant. No patient-characteristic main effect remained significant after Holm adjustment, and no model interaction was detected for age, clinician-rated item burden, major depressive episode status, or sex (all interaction-adjusted *P*>.99; Table S4 in Multimedia Appendix 3).

Among all model-specific mismatches, nonambiguous overestimation and underestimation accounted for 145 of 301 (48.2%) and 68 of 301 (22.6%), respectively, for Claude 3.5; 141 of 355 (39.7%) and 96 of 355 (27.0%) for Gemini 2.5; and 73 of 248 (29.4%) and 78 of 248 (31.5%) for GPT-4o (Table 4). Thus, even after excluding ambiguous cases, overestimation errors remained relatively more frequent than underestimation errors for Claude 3.5 and Gemini 2.5, whereas GPT-4o showed a more balanced distribution.

**Figure 2.**
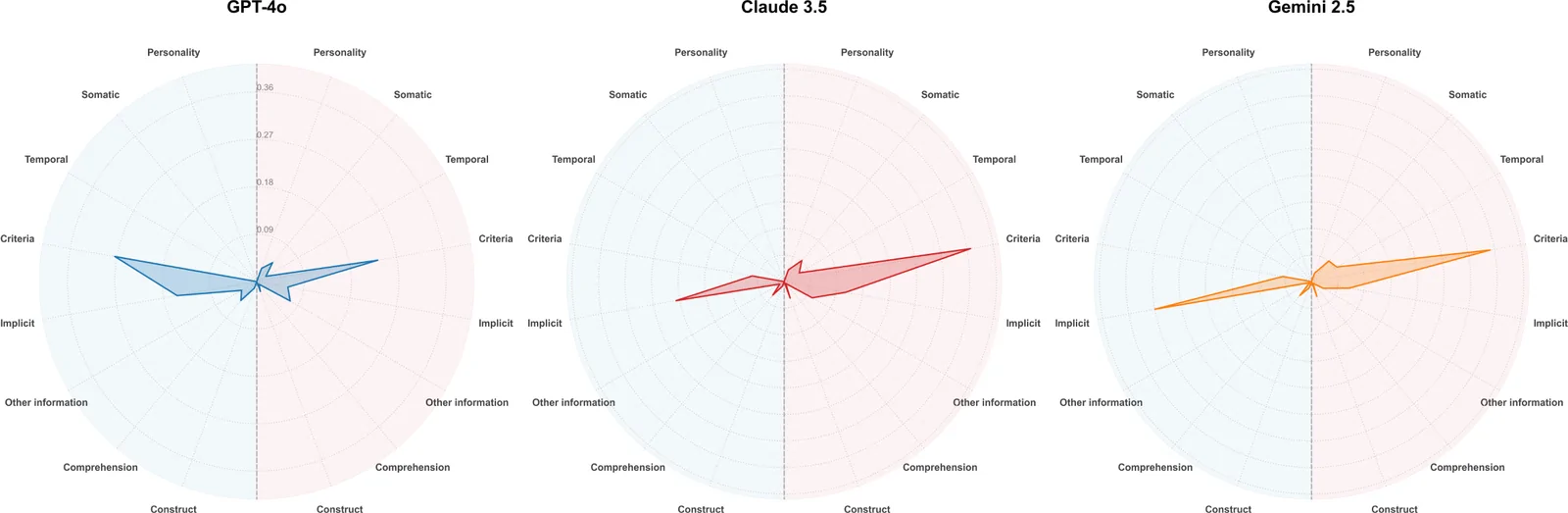
Model-wise distribution of nonambiguous error subtypes. Radar plots showing the distribution of clinician-adjudicated error subtypes among nonambiguous mismatches for GPT-4o, Claude 3.5, and Gemini 2.5. For each model, subtype proportions were renormalized to a sum of 1 after excluding ambiguous cases. The right semicircle represents overestimation subtypes, and the left semicircle represents underestimation subtypes (implicit cues, other information, comprehension, criteria application, personality attribution, somatic confounding, temporal misalignment, and construct misclassification).

Figure 2 shows the composition of nonambiguous errors by subtype, displaying the model-wise distributions after renormalizing the proportions within each model and excluding ambiguous cases. Criteria-related errors were the most frequent nonambiguous subtype across all 3 models. Claude 3.5 and Gemini 2.5 predominantly overestimated severity based on misapplied criteria, whereas GPT-4o showed a more balanced profile with relatively higher criteria-related underestimation. Implicit cue errors accounted for smaller proportions in GPT-4o than in Claude 3.5 and Gemini 2.5. Less frequent subtypes, such as errors involving other information, somatic confounding, temporal misalignment, comprehension problems, and personality attribution, each accounted for only a small proportion of nonambiguous mismatches in all models.

**Table 4.** Distribution of clinician-adjudicated error types across large language models, including ambiguous cases and direction-specific misclassifications^a^.

Direction and error type	GPT-4o, n (%)	Claude 3.5, n (%)	Gemini 2.5, n (%)
Ambiguous	97 (39.1)	88 (29.2)	118 (33.2)
Over			
Criteria^b^	35 (14.1)	76 (25.2)	81 (22.8)
Implicit cues	9 (3.6)	25 (8.3)	17 (4.8)
Comprehension	3 (1.2)	7 (2.3)	7 (2.0)
Other information	11 (4.4)	13 (4.3)	6 (1.7)
Personality^c^	4 (1.6)	5 (1.7)	4 (1.1)
Somatic^d^	7 (2.8)	11 (3.7)	12 (3.4)
Temporal^e^	3 (1.2)	7 (2.3)	13 (3.7)
Construct^f^	1 (0.4)	1 (0.3)	1 (0.3)
Under			
Criteria	41 (16.5)	13 (4.3)	13 (3.7)
Implicit cues	23 (9.3)	44 (14.6)	71 (20.0)
Comprehension	2 (0.8)	2 (0.7)	3 (0.8)
Other information	5 (2.0)	2 (0.7)	1 (0.3)
Personality	0 (0.0)	0 (0)	0 (0)
Somatic	0 (0)	0 (0)	0 (0)
Temporal	0 (0)	0 (0)	0 (0)
Construct	7 (2.8)	7 (2.3)	8 (2.3)

a Values are presented as number and percentage of all binary mismatches for each model. Percentages are calculated within each model using the total number of mismatches as the denominator, including ambiguous cases. Ambiguous cases indicate instances in which the expert adjudicator judged the transcript insufficient to determine a definite overestimation or underestimation.

b Criteria: criteria application.

c Personality: personality attribution.

d Somatic: somatic confounding.

e Temporal: temporal misalignment.

f Construct: construct misapplication.

### Reasoning Style and Association With Prediction Error

Meta-evaluation scores differed significantly between the models across all 4 dimensions (citation, structure, mapping, expansion; all *P*<.001; Table 5). Claude 3.5 had the highest citation and structure scores, Gemini 2.5 had intermediate scores, and GPT-4o had the lowest scores on these dimensions. Mapping scores were similar for Claude 3.5 and Gemini 2.5 and lower for GPT-4o, whereas expansion scores were similar for GPT-4o and Claude 3.5 and lower for Gemini 2.5 (Table 5).

In exploratory domain-adjusted cross-classified mixed-effects models, mapping was inversely associated with absolute error, whereas structure and expansion were positively associated with error across all 3 source models. Citation was positively associated with error for GPT-4o and Claude 3.5 but not for Gemini 2.5 (Table S5 in Multimedia Appendix 3). In the human-reference sensitivity analyses, coefficient directions were consistent with those observed in the primary analysis for all 4 dimensions, although statistical significance varied across dimensions and source models (Figure S2 in Multimedia Appendix 4).

**Table 5.** Meta-evaluation scores of model-generated rationales across 4 explanatory dimensions for each large language model^a^.

Dimensions	GPT-4o	Claude 3.5	Gemini 2.5	Adjusted *P* value	Kendall’s *W*
Citation	4.304	4.705	4.636	<.001	0.762
Structure	3.991	4.492	4.324	<.001	0.812
Mapping	4.066	4.295	4.293	<.001	0.540
Expansion	1.785	1.787	1.709	<.001	0.166

a Scores represent mean ratings on a 1 to 5 ordinal scale, with higher values indicating a greater presence of the corresponding feature. Adjusted *P* values indicate false discovery rate–adjusted *P* values for overall Friedman test results. Kendall’s *W* represents the effect size associated with the Friedman test.

## Discussion

This exploratory study examined how 3 LLMs reproduced psycho-oncologists’ item-level ratings from real Korean psycho-oncology interviews and how their reasoning patterns were related to errors. Across the 3 evaluated models, overall concordance with clinician ratings was high, suggesting that these models could approximate psycho-oncologists’ item-level ratings at the aggregate level. However, the structure of disagreement differed meaningfully between models. Claude 3.5 and Gemini 2.5 showed a systematic tendency toward overestimation, resulting in inflated patient-level symptom burden, whereas GPT-4o displayed a more balanced error profile without such inflation. Expert adjudication indicated that a substantial proportion of disagreements stemmed from the ambiguity inherent in the transcripts, whereas clear misclassifications were dominated by the misapplication of clinical thresholds. Exploratory meta-evaluation suggested that lower mapping and higher structure or expansion scores may accompany larger rating errors.

Evidence of LLM performance in clinical medicine has been largely generated from clinician-authored records or other proxy texts [9]. Although a recent study indicated that LLMs can detect depression and anxiety in patients with chronic disease contexts [22], relatively few studies have evaluated models using semistructured psychiatric interviews, often in narrower settings [17]. The present exploratory findings extend existing evidence by showing high aggregate concordance across all 3 evaluated models for a heterogeneous set of 39 symptoms and interference items using real-world psycho-oncology interviews conducted in a non-English clinical setting. By directly evaluating original Korean interview transcripts rather than translated or proxy texts, this study found high aggregate concordance between the 3 evaluated LLMs and psycho-oncologists’ item-level ratings in this Korean-language psycho-oncology sample. While these findings should be interpreted as exploratory evidence of model behavior rather than as definitive validation for clinical deployment, prospective, workflow-specific studies may examine whether transcript-derived item-level ratings could serve as supplementary information for clinician-supervised postinterview review, with clinicians retaining responsibility for interpretation and clinical decisions. The findings also contribute to addressing gaps associated with the English-dominant and Western-centric nature of contemporary LLM training and benchmarking [14,23]. Moreover, the clinician-adjudicated error review and structured meta-evaluation of model rationales provided complementary exploratory analyses of how models diverged from clinician judgment. These analyses suggest that models with similar aggregate agreement may still differ in directional bias, recurrent error patterns, and explanatory behavior, supporting the need to examine model performance beyond single-number summary metrics [10].

This study focused on item-level symptom ratings to better align with the requirements of granular symptom monitoring in oncologic care [24]. From a model-evaluation perspective, decomposing assessment into individual items offers a more transparent and reviewable approach than asking a model to assign a diagnosis directly. Item-level ratings enable disagreements to be localized and may, in principle, be combined using prespecified external rules, consistent with structured scoresheet approaches [25]. This modular framework supports evaluating item-level agreement as a distinct component of LLM-based psychiatric assessment and motivates future research examining whether such ratings can contribute to reliable syndrome-level classification.

Although the 3 LLMs exhibited broadly similar overall agreement with the psycho-oncologists, they differed in the distribution and direction of errors. Previous LLM-based depression-screening research has likewise reported variation in performance across models and symptom items [26]. In our study, Claude 3.5 had the highest sensitivity and NPV point estimates but also generated more false-positive classifications, while Claude 3.5 and Gemini 2.5 yielded higher patient-level symptom counts than the clinician ratings. In contrast, GPT-4o had the highest PPV and did not show a statistically significant increase in patient-level symptom counts. These findings suggest that aggregate agreement alone may obscure differences in the balance between missed and overidentified symptoms. If replicated across model versions and independent samples, such differences may help clarify the relevance of particular error profiles across assessment contexts.

In this study, clinician adjudication helped distinguish definite model errors from disagreements arising from incomplete, indirect, or context-dependent transcript evidence. Such ambiguity may reflect a characteristic of semistructured psychiatric interviews and may limit the extent to which definitive judgments can be made from interview transcripts alone [11]. Within the subset of disagreements judged to represent definite misclassification, the most common mechanism across the models was the misapplication of severity thresholds. This pattern is potentially important because threshold errors shift the decision boundary and may preferentially increase false positives or false negatives. Other errors were less frequent but particularly salient in psycho-oncology, where treatment-related neurovegetative symptoms closely mimic affective distress [1]. Consistent with prior studies, the tendency to interpret somatic symptoms as depressive indicators represents a common challenge in AI-based clinical reasoning [27]. The clinician-adjudicated taxonomy provides a structured account of recurrent disagreement mechanisms and may help identify priorities for model refinement. The predominance of threshold errors suggests that future prompt-development studies should examine whether clearer operationalization of severity cutoffs and stronger anchoring to the target time window improve rating consistency. Similarly, the occurrence of somatic confounding highlights the importance of evaluating strategies that distinguish cancer- or treatment-related neurovegetative symptoms from psychological distress. In this way, the taxonomy offers an empirically grounded framework for developing and testing targeted error-mitigation strategies, consistent with prior work on prompt-based mitigation in medical LLMs [28].

Rationale characteristics also differed across models. Claude 3.5 produced more structured and citation-dense justifications, whereas GPT-4o was lower on these dimensions and Gemini 2.5 generally showed an intermediate profile. Exploratory mixed-effects analyses suggested that more structured or evidence-rich rationales did not necessarily correspond to closer agreement with psychiatrist ratings. This observation is broadly consistent with prior studies showing that eliciting explicit reasoning may improve interpretability without reliably improving task performance [29,30]. The analyses also suggested that weaker mapping and greater interpretive expansion may accompany larger absolute error across source models. These observations support further examination of prompting strategies that more explicitly connect transcript evidence with scoring criteria while discouraging unsupported extrapolation. However, greater expansion may also be a marker of difficult or ambiguous cases that elicit both greater interpretive extrapolation and greater model-clinician disagreement, rather than a cause of error. Because the present study evaluated observable rationale characteristics rather than the models’ internal decision processes, it does not establish whether the generated rationales reflected the processes underlying the final outputs [31].

Several limitations should be noted. First, this exploratory study included 101 patients from Korean-language psycho-oncology settings and predominantly represented women and patients with breast cancer, with limited representation of survivorship settings. The 3931 item-level ratings were clustered within these 101 interviews and did not constitute independent patient-level observations, limiting subgroup analyses. Accordingly, the generalizability of the findings to male patients, other cancer types, survivorship populations, non-Korean linguistic and cultural contexts, or nononcologic psychiatric populations should be examined in larger, more diverse, and independent cohorts. Second, although interrater reliability among psychiatrists was high, the substantial proportion of ambiguous cases highlights that some degree of interpretive variability is intrinsic to semistructured psychiatric interviews. Third, the analyses were based on text transcripts, whereas clinicians had access to nonverbal cues integral to mental status assessment. Fourth, the use of time-stamped clinician labels enables item-specific segmentation, which may represent a more favorable setting than typical transcript- or STT-based workflows. Fifth, case-level adjudication of the full mismatch set was performed by 1 primary psychiatrist. While a second independent reviewer showed high agreement for the ambiguous-versus-definite distinction in a stratified audit of 150 cases (Cohen κ=0.82), residual subjectivity remains possible. Moreover, although no broad clinical-domain or patient subgroup heterogeneity was detected, ambiguous adjudications constituted a substantial proportion of mismatches and differed between GPT-4o and Claude 3.5. Sixth, in the 10-patient subset, agreement between the mean automated meta-evaluation scores and human ratings ranged from ICC (A,1)=0.646 to 0.822 across dimensions, with moderate reliability for citation and structure. This residual measurement uncertainty and the possibility of model-family-specific bias despite the multievaluator approach represent important limitations, and the findings based on the automated meta-evaluation scores should therefore be interpreted as exploratory. Finally, the evaluation was limited to specific model versions available at the time of analysis; future updates or fine-tuned variants may exhibit different error profiles.

In conclusion, this exploratory clinician-benchmarked evaluation showed that contemporary LLMs could approximate symptom-level assessment in a Korean psycho-oncology sample composed predominantly of women and patients with breast cancer. Despite high aggregate concordance, the models differed in directional bias and disagreement patterns, suggesting that similar overall performance may obscure potentially meaningful differences in their observed error profiles. These findings require further validation in larger, more diverse, and independent cohorts.

## Supplementary material

10.2196/91405 Multimedia Appendix 1 Zero-shot prompt for large language model–based symptom evaluation, reliability analyses of automated meta-evaluation scores against human reference ratings, and meta-evaluation prompts and scoring rubrics.

10.2196/91405 Multimedia Appendix 2 Deidentified examples of clinician–large language model disagreements adjudicated as ambiguous.

10.2196/91405 Multimedia Appendix 3 Ambiguity-excluded binary screening performance, ambiguous-disagreement distributions and regression analyses, and associations between meta-evaluation scores and absolute rating error.

10.2196/91405 Multimedia Appendix 4 Ordinal confusion matrices comparing model-generated and clinician symptom-severity ratings and domain-adjusted mixed-effects estimates of associations between automated and human-reference meta-evaluation scores and absolute rating error.

10.2196/91405 Checklist 1 TRIPOD-LLM checklist.

## Abbreviations

GPT: generative pretrained transformer
ICC: intraclass correlation coefficient
LLM: large language model
NPV: negative predictive value
PPV: positive predictive value
QWK: quadratic weighted kappa
STT: speech-to-text
TRIPOD-LLM: Transparent Reporting of a Multivariable Prediction Model for Individual Prognosis or Diagnosis–Large Language Model
