# Supplementary material for: Large Language Models for Distress Rating in Korean Psycho-Oncology Interviews: Exploratory Clinician-Benchmarked Evaluation Study
Source: J Med Internet Res. 2026 Sep 9;28:e91405. doi: 10.2196/91405 (PMC13557317; doi:10.2196/91405)
Supplement: Supplementary file 2 [file jmir-v28-e91405-s001.docx]

**Multimedia Appendix 1. Supplementary Methods**

## **Supplementary Methods 1. Zero-shot prompt used for large language model evaluation (English translation).**

System prompt

\## Instruction

- You are a board-certified psychiatrist with expertise in psycho-oncology.

- The provided script is a dialogue between a psychiatrist and a cancer patient for the purpose of distress management. Evaluate each item and generate outputs according to the rules below.

\## Rule

A. Evaluation principles

1. Analyze the patient’s statements in the script to assess the patient’s condition over the past 1–2 weeks as it relates to each item.

2. Focus on statements directly related to the evaluation item; family history or background information should be used only as supplementary context when necessary.

3. Conduct the evaluation based on the [Item Tag], while taking the overall context into account.

B. Scoring criteria

4. Assign scores according to the “4-Point Psychiatric Rating Scale” below, and enter a number from 0 to 3 as a string (e.g., “2”) in evaluation_value.

5. Patient expressions should be evaluated based on frequency, duration, and functional impact; in particular, the distinction between scores 0–1 and 2–3 is clinically critical.

6. If judgment is difficult, avoid speculation and conservatively choose a lower score (0–1).

7. In “evaluation_reason,” clearly describe the rationale for the assigned score by comparing the patient’s responses with the rating criteria.

C. Item tags

8. The script contains tags indicating item sections.

9. Tags consist of a start tag in the form [ItemGroup] and an end tag in the form [ItemGroup|ItemNumber].

10. The dialogue between a start tag and an end tag corresponds to the response content for that item.

11. Multiple end tags may sequentially appear under a single start tag.

D. Insufficient statements and follow-up handling

12. If there is no statement at all related to an evaluation item, evaluation_value must be set to “0”.

13. If the patient’s condition has changed across multiple time periods, the evaluation should reflect the patient’s status over the “most recent 1–2 weeks.”

\### 4-Point Psychiatric Rating Scale

| Score | Frequency (per week) | Daily duration | Functional impact | Clinical interpretation | Example expressions |

|------|----------------------|----------------|-------------------|------------------------|---------------------|

| 0 | None, 0–1 times/week | None, momentary | No impact | Normal / negligible | “Not at all,” “Never experienced it” |

| 1 | Rarely, 1–2 times/week | Transient | Minimal or no impact | Mild symptoms | “Occasionally,” “It happened for about a day” |

| 2 | Often, 3–4 times/week | Part of the day | Some interference | Moderate symptoms (e.g., reduced concentration, increased fatigue, decreased interest leading to passive participation) | “Quite often,” “It lasted for several days” |

| 3 | Almost daily, 5–7 times/week | Most of the day or all day | Unable to function (e.g., absence from school/work, social withdrawal, disruption of basic functions such as sleep or eating) | Severe symptoms, intervention needed | “Every day,” “It lasted all day” |

\## Script information

This script is an STT (speech-to-text) transcription of a spoken conversation. Make judgments while considering the following possible errors:

| Error type | Interpretation guideline |

|----------------|---------------------------|

| Similar-sounding word errors | Infer words from context; if ambiguous, assign a lower score |

| Excessive personal details | Ignore if not directly related to the evaluation item |

| Ungrammatical/repetitive speech | When emotional expressions are repeated, focus on the core meaning (e.g., repeated “It’s hard” → may indicate depression) |

| Indirect expressions | Even without explicit statements, repetitive or persistent emotional expressions should be reflected in the evaluation |

\## OUTPUT FORMAT RULES

- **Strictly prohibited:** Do not use backticks (```), quotation marks (''' or ""), or any other kind of code block markers in the response.

- **Pure JSON:** The output must be strictly in pure JSON format. Do NOT include any explanatory text, introduction, conclusion, or markdown formatting outside the JSON.

- **Exact start and end:** The response must start with an opening brace `{` and end with a closing brace `}`. No spaces, line breaks, or any other characters are allowed before or after these braces.

- **No Unicode artifacts:** Do not include unnecessary Unicode characters such as non-breaking spaces (`\xa0`).

- **JSON internal rules:** All keys and string values must be enclosed in double quotation marks (`"`). Never use single quotation marks (`'`). Numbers, booleans (true/false), and null values must not be enclosed in quotation marks.

User prompt

\## Script

{{fullscripts}}

\## [ItemTag]:Item

1. MDEA4-1-1: If you felt it was difficult to fall asleep over the past 1–2 weeks, how severe was it?

2. MDEA4-1-2: If you felt that you woke up frequently or could not sleep deeply over the past 1–2 weeks, how severe was it?

3. MDEA4-1-3: If you woke up too early in the morning over the past 1–2 weeks, how severe was it?

4. MDEA4-2: If you slept too much or felt excessively sleepy over the past 1–2 weeks, how severe was it?

5. MDEA6: If you experienced persistent fatigue or lethargy over the past 1–2 weeks, how severe was it?

6. MDEA3-1: If you felt that your appetite had decreased compared to usual over the past 1–2 weeks, how severe was it?

7. MDEA3-2: If you felt that your appetite had increased excessively compared to usual over the past 1–2 weeks, how severe was it?

8. MDEA1: How has your mood been over the past 1–2 weeks? Have you felt sad, empty, or depressed?

9. MDEA1-1: Over the past 1–2 weeks, have you been easily hurt emotionally or often felt hurt or disappointed?

10. MDEA1-2: Over the past 1–2 weeks, have you been easily irritated or prone to anger?

11. MDEA1-3: Over the past 1–2 weeks, how often have you experienced marked mood swings or emotional instability?

12. MDEA2: How is your interest or enjoyment? Compared to usual, has your interest in things decreased, or have activities you used to enjoy become less pleasurable?

13. MDEA5-1: Over the past 1–2 weeks, how severe was your feeling of restlessness or agitation?

14. MDEA5-2: Over the past 1–2 weeks, have you felt that your speech or movements have slowed down? To what extent?

15. MDEA8-1: If you felt that your concentration was poor over the past 1–2 weeks, how severe was it?

16. MDEA8-2: If you felt burdened or found it difficult to make decisions over the past 1–2 weeks, how severe was it?

17. MDEA8-3: If you felt that your thinking was slowed or that your mind was not working well over the past 1–2 weeks, how severe was it?

18. MDEA8-5: If you experienced discomfort due to memory decline over the past 1–2 weeks, how severe was it?

19. MDEA7-1: If you felt useless over the past 1–2 weeks, how severe was it?

20. MDEA7-2: If you felt guilty over the past 1–2 weeks, how severe was it?

21. MDEA9-1: If you frequently thought about death over the past 1–2 weeks, how severe was it?

22. MDEA9-2: If you felt like giving up on cancer treatment over the past 1–2 weeks, how severe was it?

23. MDEA9-3: If you felt that you would rather die quickly over the past 1–2 weeks, how severe was it?

24. MDEA9-4: Have you recently had thoughts about committing suicide?

25. MDEA9-5: Have you ever made a specific plan for how to commit suicide?

26. MDEA9-6: Have you ever actually attempted suicide?

27. ANX: If you felt anxious or tense over the past 1–2 weeks, how severe was it?

28. FoP: If you have fear about cancer recurrence or progression, how severe was it?

29. Burden: If you felt that you were a burden to your family or others due to cancer over the past 1–2 weeks, how severe was it?

30. IMPACT1: How distressing have these psychological symptoms (anxiety, depression, guilt, etc.) been for you?

31. IMPACT2: If these psychological symptoms (anxiety, depression, guilt, etc.) caused difficulties in your relationships with family, colleagues, or friends, how severe was it?

32. IMPACT3: If these psychological symptoms (anxiety, depression, guilt, etc.) caused difficulties in performing roles at home, school, or work, how severe was it?

33. SS1: If you experienced nausea or a feeling like you might vomit over the past 1–2 weeks, how severe was it?

34. SS2: If you experienced palpitations, chest tightness, or shortness of breath over the past 1–2 weeks, how severe was it?

35. SS3: If you experienced facial flushing or excessive sweating over the past 1–2 weeks, how severe was it?

36. SS4: If you experienced pain over the past 1–2 weeks, how severe was it?

37. IMPACT4: How distressing did you personally find these physical symptoms (nausea, palpitations, flushing, etc.)?

38. IMPACT5: If these physical symptoms (nausea, palpitations, flushing, etc.) caused difficulties in your relationships with family, colleagues, or friends, how severe was it?

39. IMPACT6: If these physical symptoms (nausea, palpitations, flushing, etc.) caused difficulties in performing daily roles at school, home, or work, how severe was it?

\## OUTPUT FORMAT - Follow Strictly (BUT DO NOT WRAP THE JSON CODES IN JSON MARKERS)

{

"MDEA4-1-1": {

"evaluation_value": "",

"evaluation_reason": ""

},

"MDEA4-1-2": {

"evaluation_value": "",

"evaluation_reason": ""

},

……

"IMPACT6": {

"evaluation_value": "",

"evaluation_reason": ""

}

}

## **Supplementary Methods 2. Reliability of LLM-Based Meta-Evaluation Scores Against Human Reference Ratings**

**Reliability Dataset and Score Construction**

Human reference ratings were provided by the primary evaluator for a subset of 10 patients. The reliability dataset contained 1,170 source-model/item observations: 39 clinical items for each patient and three source rationale models (GPT-4o, Claude 3.5, and Gemini 2.5), corresponding to 390 observations per source model. GPT-5.4, Claude Sonnet 4.6, and Gemini Pro 3.1 independently evaluated each rationale using the same four-dimensional rubric. For each rationale and dimension, the continuous arithmetic mean of the three independently assigned scores was compared with the human reference rating.

**Descriptive Agreement**

Overall descriptive agreement is presented in Table S1. Mean differences between the LLM-based mean scores and human reference ratings were small for Structure (0.003), Citation (0.068), Mapping (-0.082), and Expansion (-0.112). Mean absolute differences ranged from 0.332 for Mapping to 0.409 for Structure.

Table S1. Overall descriptive agreement between LLM-based meta-evaluation scores and human reference ratings

| Dimension | Mean human reference | Mean LLM-based score | Mean absolute difference |
| --- | --- | --- | --- |
| Citation | 4.478 | 4.546 | 0.348 |
| Structure | 4.299 | 4.302 | 0.409 |
| Mapping | 4.323 | 4.241 | 0.332 |
| Expansion | 1.882 | 1.770 | 0.344 |

**Overall and Source-Model-Specific Reliability**

Agreement was quantified using two-way mixed-effects, absolute-agreement, single-measure intraclass correlation coefficients [ICC(A,1)]. Overall ICCs pooled the observations from all three source rationale models, whereas source-model-specific ICCs were calculated separately for GPT-4o-, Claude 3.5-, and Gemini 2.5-generated rationales. Uncertainty was estimated using 2,000 patient-cluster bootstrap resamples, with all observations from a sampled patient retained within each resample.

Overall and source-model-specific ICCs are presented in Table S2. Overall ICC(A,1) values were 0.669 for Citation, 0.646 for Structure, 0.805 for Mapping, and 0.822 for Expansion. Source-model-specific ICCs ranged from 0.565 to 0.695 for Citation, 0.573 to 0.603 for Structure, 0.777 to 0.818 for Mapping, and 0.798 to 0.848 for Expansion. The source model with the numerically highest ICC varied across dimensions, with no consistent ordering of the three source models.

Table S2. Overall and source-model-specific agreement with human reference ratings

| Model | Citation | Structure | Mapping | Expansion |
| --- | --- | --- | --- | --- |
| Overall | 0.669 (0.611-0.717) | 0.646 (0.583-0.701) | 0.805 (0.755-0.842) | 0.822 (0.789-0.845) |
| GPT | 0.565 (0.428-0.695) | 0.603 (0.513-0.683) | 0.818 (0.768-0.858) | 0.798 (0.735-0.837) |
| Claude | 0.590 (0.492-0.688) | 0.573 (0.451-0.684) | 0.806 (0.744-0.855) | 0.848 (0.822-0.877) |
| Gemini | 0.695 (0.623-0.768) | 0.601 (0.507-0.681) | 0.777 (0.683-0.842) | 0.816 (0.776-0.847) |

Values are ICC(A,1) estimates with 95% patient-cluster bootstrap confidence intervals.

**Source-Model Heterogeneity**

Differences among the three dependent source-model-specific ICCs were evaluated for each dimension using a 2-df Wald test. Paired patient-cluster bootstrap replicates were used to estimate the joint covariance matrix of the three ICC estimates and construct a null-centered reference distribution for the Wald statistic. The four bootstrap-calibrated *P* values were adjusted using the Holm procedure.

Comparisons of the three source-model-specific ICCs are presented in Table S3. The unadjusted bootstrap *P* values were .031 for Citation and .046 for Expansion, but none of the four comparison tests was statistically significant after Holm adjustment (all adjusted *P*≥.126). Thus, the analysis did not identify statistically significant heterogeneity in ICC according to the source rationale model after accounting for multiplicity.

Table S3. Comparison tests of source-model-specific ICC differences

| Dimension | Wald statistic, *W* | Unadjusted *P* | Holm-adjusted *P* |
| --- | --- | --- | --- |
| Citation | 6.718 | .031 | .126 |
| Structure | 0.214 | .905 | 1.000 |
| Mapping | 1.141 | .573 | 1.000 |
| Expansion | 6.025 | .046 | .138 |

All tests had 2 df.

## **Supplementary Methods 3. Prompts for meta-evaluation of large language model–generated symptom evaluation rationales (English translation).**

\# System Prompt

You are a board-certified psychiatrist with expertise in psycho-oncology and clinical rating-scale evaluation.

Your task is not to re-rate the patient directly. Your task is to evaluate the quality and traceability of an AI-generated symptom evaluation rationale.

This meta-evaluation must be performed as an independent judgment task. Do not replace the rubric with deterministic scoring rules, keyword matching, automated approximations, or precomputed scores. For each item, read the interview script and the AI-generated rationale and assign scores based on the rubric below.

For each item, compare:

1. The interview script.

2. The target symptom item and tag.

3. The AI-assigned score.

4. The AI-provided evaluation reason.

Assess whether the AI's rationale gives enough evidence and reasoning for a human reviewer to understand why that score was assigned.

Do not reward verbosity alone. A long rationale is good only when it improves Citation (Evidence_Anchoring), Structure (Reasoning_Transparency), and Mapping (Rule_Mapping).

Do not penalize a rationale merely because there was no symptom-related utterance in the script. If the full script truly contains no relevant utterance for the item, and the AI accurately states that absence or insufficiency of evidence, that can receive a high Citation (Evidence_Anchoring) score.

\# User Prompt Template

```text

\# Instruction: Meta-evaluator for AI symptom-evaluation rationales

\## Evaluation Goal

Evaluate the quality of the AI-generated rationale for each symptom-rating item.

You must score four axes:

1. Citation (Evidence_Anchoring)

2. Structure (Reasoning_Transparency)

3. Mapping (Rule_Mapping)

4. Expansion (Inferential_Distance)

You must first classify Evidence_Status. Evidence_Status describes the relationship between the script evidence and the target item, and it informs Structure (Reasoning_Transparency), Mapping (Rule_Mapping), Expansion (Inferential_Distance), and problem flags. Citation (Evidence_Anchoring) should focus on whether the AI accurately and specifically anchors its stated evidence in the actual script; do not use Citation primarily to judge whether that evidence is sufficient for the assigned score.

\## Clinical Rating Context

The original AI rated each item on a 0-3 psychiatric symptom scale:

| Score | Frequency | Duration | Functional impact | Interpretation |

|---|---|---|---|---|

| 0 | None or negligible | None/momentary | No impact | Normal or absent |

| 1 | Rare/mild | Transient | Minimal or no impact | Mild |

| 2 | Often/moderate | Part of the day or several days | Some interference | Moderate |

| 3 | Almost daily/severe | Most/all day | Unable to function or severe disruption | Severe |

The intended rating window is the most recent 1-2 weeks unless the item explicitly says otherwise.

\## Step 1. Classify Evidence_Status

For each tag, inspect the full interview script and classify one of the following:

- direct_positive_evidence: The script contains a directly relevant utterance indicating the symptom is present.

- direct_negative_evidence: The script contains a directly relevant utterance denying the symptom or its impact.

- no_relevant_evidence: The full script contains no utterance meaningfully related to this item.

- cross_tag_evidence_only: Relevant evidence exists in an adjacent or different construct, but it does not directly address the target symptom, behavior, or its clinically inclusive construct family.

- ambiguous_or_stt_unclear: The utterance is unclear because of ambiguous wording, context, or transcription/STT issues.

Important: no_relevant_evidence is not automatically poor citation. If the AI accurately states that there is no relevant mention or insufficient evidence, Citation (Evidence_Anchoring) may be high.

Classify Evidence_Status by semantic relevance to the target item, not merely by where the utterance appears in the interview or which nearby tag it resembles. When evidence comes from an adjacent item within the same clinical construct family, classify it according to semantic relevance rather than tag identity alone. For example, explicit denial of suicidal ideation may provide relevant negative evidence for suicidal plan or attempt within the current rating window, if there is no contradictory evidence.

However, keep Citation separate from item-score appropriateness. If the rationale accurately states the broader evidence, such as "the patient denied suicidal ideation," Citation may be high even when the target item is narrower, such as suicidal plan or attempt. If the rationale converts broader construct-level evidence into a narrower item-specific statement that the script did not say, such as changing "no suicidal ideation" into "no suicidal plan," lower Citation for over-specific or overstated anchoring. Evaluate whether the broader evidence is sufficient for the target item and assigned score under Mapping, Expansion, and problem flags.

\## Axis 1. Citation (Evidence_Anchoring) (1-5)

Definition: How specifically and accurately the AI rationale anchors its stated evidence in the actual interview script, including accurate handling of evidence absence.

Citation is primarily a textual anchoring axis. It evaluates whether the rationale quotes, paraphrases, summarizes, or states absence of script evidence accurately. It does not primarily judge whether the cited evidence is sufficient or appropriate for the target item score; judge that under Structure, Mapping, Expansion, and problem flags.

Score using the rule that matches Evidence_Status.

\### If Evidence_Status is direct_positive_evidence or direct_negative_evidence

| Score | Criterion |

|---|---|

| 1 | The rationale is not traceable to the script, fabricates evidence, materially changes the utterance, or says the opposite of the script. |

| 2 | The rationale is broadly related to the script but too generic or over-specific to verify as the stated evidence. |

| 3 | The rationale captures the core meaning but lacks concrete wording, timing, frequency, intensity, denial, impact, or context needed to verify the stated evidence. |

| 4 | The rationale gives a close paraphrase or specific script-based evidence that is clearly verifiable, but does not quote or nearly identically preserve all available clinically important anchors. |

| 5 | The rationale directly quotes or nearly identically paraphrases the relevant utterance, preserving the stated evidence and important anchors such as timing, frequency, intensity, denial, or functional impact. For simple direct-denial items, preserving the symptom/construct and denial polarity may be sufficient if no other anchor is available. |

\### If Evidence_Status is no_relevant_evidence

| Score | Criterion |

|---|---|

| 1 | The AI invents a symptom, denial, or direct evidence that is not present in the script. |

| 2 | The AI gives a conclusion without acknowledging the absence of relevant evidence. |

| 3 | The AI vaguely implies non-reporting, such as "did not complain," but does not make clear that the script lacks relevant evidence. |

| 4 | The AI states that there is no direct or sufficient evidence for the item. |

| 5 | The AI explicitly states that the full script contains no relevant mention for this item and therefore uses a conservative/0 score. |

\### If Evidence_Status is cross_tag_evidence_only or ambiguous_or_stt_unclear

| Score | Criterion |

|---|---|

| 1 | The AI fabricates, reverses, or materially misstates the indirect/ambiguous evidence, or presents a narrower item-specific utterance that the script did not contain. |

| 2 | The AI refers to adjacent or ambiguous evidence but states it too generically or too specifically to verify from the script. |

| 3 | The AI captures the broad meaning of the adjacent/ambiguous evidence, but the exact wording, source, or limitation is unclear. |

| 4 | The AI accurately paraphrases the adjacent/ambiguous evidence and does not materially overstate what the script said. |

| 5 | The AI directly quotes or nearly identically paraphrases the adjacent/ambiguous evidence and preserves the limitation, such as broader construct-level evidence, cross-tag location, or ambiguous wording. |

\### Citation 4 vs 5 calibration examples

Use these examples only to calibrate the Citation boundary. Do not turn them into keyword rules.

Example 1: Direct positive evidence with concrete anchors

- Script: "These days I wake up almost every day around 3-4 a.m., and it is hard to fall asleep again."

- AI rationale A: "The patient wakes up often."

- Citation = 3, because the core meaning is present but timing, frequency, and difficulty returning to sleep are missing.

- AI rationale B: "The patient wakes up often recently and has difficulty returning to sleep."

- Citation = 4, because the rationale is clearly traceable and clinically accurate, but it omits available anchors such as "almost every day" and "around 3-4 a.m."

- AI rationale C: "The patient wakes up almost every day around 3-4 a.m. and has difficulty returning to sleep."

- Citation = 5, because it faithfully preserves the clinically important anchors without requiring a verbatim quote.

Example 2: Simple direct denial

- Script: "I do not feel restless or unable to sit still."

- AI rationale A: "The patient denied restlessness or psychomotor agitation."

- Citation = 5, because for a simple direct-denial item, preserving the symptom/construct and denial polarity is sufficient when no additional anchor is available.

- AI rationale B: "The patient denied anxiety symptoms."

- Citation = 3 or 4, because the denial is broadly related but less item-specific; judge whether it remains clearly traceable to the target item.

Example 3: Broader construct-level evidence

- Target item: suicidal plan

- Script: "I do not have thoughts that I want to die."

- AI rationale A: "The patient denied suicidal ideation."

- Citation = 5, because the rationale accurately anchors the broader construct-level evidence that was actually stated.

- AI rationale B: "The patient denied a suicidal plan."

- Citation = 3 or lower, because the script did not directly mention plan; the rationale converts broad evidence into a narrower item-specific denial.

- Mapping and Expansion should then judge whether the broader denial of suicidal ideation is sufficient to support the assigned score for suicidal plan.

\## Axis 2. Structure (Reasoning_Transparency) (1-5)

Definition: How easy it is for a human reviewer to reconstruct the AI's reasoning from script evidence to interpretation to score.

This axis is meant to capture whether the rationale is reviewable and traceable. Structure should evaluate whether the rationale makes the reasoning path transparent, including whether it separates script evidence from interpretation when the evidence is broad, adjacent, indirect, ambiguous, or not item-specific.

Structure evaluates whether the rationale makes the reasoning path visible, not whether the assigned score is clinically correct. Judge the appropriateness or sufficiency of the cited rating dimensions under Mapping.

Use these anchors:

- 5 = fully reconstructable: the complete path from evidence or evidence absence to target item, interpretation, and score level is visible.

- 4 = clear but compressed: the reasoning path is followable, but one relevant anchor, caveat, or scope distinction is abbreviated.

- 3 = plausible but partly implicit: evidence and conclusion are present, but the reviewer must supply an important bridge.

| Score | Criterion |

|---|---|

| 1 | Result only, or near-result-only; the reason does not show how the score was derived. |

| 2 | Minimal rationale with little evidence or interpretation; the reviewer must infer most of the reasoning. |

| 3 | Evidence and conclusion are present, but an important bridge is implicit, such as target-item scope, time window, direct versus broader evidence, or why this severity level was chosen. |

| 4 | Evidence, interpretation, and score conclusion are explicit enough to follow. For score 0, this can include item-specific direct denial or clearly item-specific absence linked to the 0 conclusion. For scores above 0, at least one score-level anchor should be visible, such as frequency, duration, intensity, timing, or functional impact. |

| 5 | The rationale is fully reconstructable: it identifies relevant evidence or evidence absence, explains the clinical interpretation, handles direct versus broader/adjacent evidence transparently when relevant, and connects the target item to the exact score level using the key available rating dimensions. |

Application notes:

- Rationale length alone should not determine Structure; score only how visible and reconstructable the reasoning path is.

- If broader, adjacent, or non-item-specific evidence is used, the rationale should explain the bridge to the target item. Without that bridge, Structure is usually around 3 even if Citation is high.

- For broader/adjacent evidence to receive Structure 5, both the bridge and the limitation should be explicit.

\## Axis 3. Mapping (Rule_Mapping) (1-5)

Definition: Whether the AI's interpretation maps to the assigned 0-3 score in a rule-consistent and reproducible way.

Mapping evaluates how explicitly and reproducibly the AI-provided evaluation_reason connects the assigned score to the relevant rating dimensions and target item. Do not give high Mapping scores merely because the score can be justified by the script; the rationale itself should make the score-to-rule connection sufficiently clear for a reviewer.

Mapping is the primary axis for judging whether accurately cited evidence is sufficient and appropriate for the target item score. A rationale can have high Citation but low Mapping if it accurately quotes or paraphrases the script yet uses that evidence poorly for the target item, rating window, severity level, frequency, duration, or functional-impact interpretation.

Apply the mapping rule conditionally.

\### If the AI assigned score 0

| Score | Criterion |

|---|---|

| 1 | Score 0 contradicts clear symptom evidence. |

| 2 | Score 0 is weakly supported, depends on unacknowledged assumptions, or uses broader/adjacent evidence without explaining why it supports absence of the target item. |

| 3 | Score 0 is plausible but the rationale does not clearly distinguish direct denial, no evidence, current resolution, or broader construct-level negative evidence. |

| 4 | Score 0 is clearly linked to direct denial, no relevant evidence, clinically negligible symptoms, or broader construct-level negative evidence with a reasonable conservative implication. |

| 5 | Score 0 is reproducibly justified by explicit direct denial/no relevant evidence or clearly explained broader construct-level negative evidence, plus correct handling of timing and tag scope. |

\### If the AI assigned score 1

| Score | Criterion |

|---|---|

| 1 | Score 1 contradicts the evidence or uses the wrong tag. |

| 2 | Mild score is asserted with little support, or the cited evidence is not clearly appropriate for the target item. |

| 3 | Mild score is plausible, but target-item relevance, frequency/duration/impact, or uncertainty handling is incomplete. |

| 4 | Mild score is supported by target-relevant low intensity, low frequency, short duration, minimal impact, or explicit uncertainty. |

| 5 | Mild score is reproducibly justified using target-relevant evidence, the relevant rating dimensions, and conservative handling of uncertainty. |

\### If the AI assigned score 2 or 3

| Score | Criterion |

|---|---|

| 1 | Moderate/severe score contradicts the evidence or uses the wrong tag. |

| 2 | Moderate/severe score is asserted without sufficient target-relevant or rule-based support. |

| 3 | Some linkage exists, but target-item relevance is incomplete or fewer than two of frequency, duration, and functional impact are specified. |

| 4 | Target-relevant evidence and at least two relevant rating dimensions are specified and support the score, such as frequency, duration, severity, or functional impact. |

| 5 | Target-relevant evidence plus frequency, duration, and functional impact are all specified or clearly linked in the rationale, making the score reproducible. |

\## Axis 4. Expansion (Inferential_Distance) (1-5)

Definition: The degree of inferential distance between the available script evidence and the AI's assigned score/rationale.

Expansion captures how much interpretation is required to move from the script evidence to the AI's assigned score and stated rationale. Do not score this axis based only on how long or elaborate the rationale is. A brief rationale may still receive a higher Expansion score if the assigned score requires substantial clinical, contextual, cross-tag, severity, timing, frequency, duration, or functional-impact inference beyond explicit utterances.

Do not infer the AI's hidden reasoning process. Instead, judge how much inference would be needed for a human reviewer to justify the assigned score and stated rationale from the available script evidence.

This is primarily a descriptive axis, not a quality axis. Higher does not automatically mean better. Lower does not automatically mean worse.

| Score | Criterion |

|---|---|

| 1 | Minimal inferential distance: the assigned score/rationale follows directly from explicit denial, explicit symptom statement that directly supports both symptom presence and the assigned severity/impact without additional inference, or clear absence of relevant evidence. |

| 2 | Low inferential distance: the score/rationale requires only conservative summarization, straightforward absence handling, or a small inference from explicit utterances. |

| 3 | Moderate inferential distance: direct symptom evidence exists, but severity, timing, frequency, duration, or impact must be partly inferred to justify the score. |

| 4 | High inferential distance: the score/rationale substantially depends on indirect cues, local context, cross-tag evidence, or clinically meaningful inference beyond the target utterance. |

| 5 | Very high inferential distance: the score/rationale requires broad clinical inference, substantial time-window inference, or moderate/severe scoring from sparse, indirect, ambiguous, or weakly specified evidence. |

Important: Score Expansion by how much inference is required, not by whether that inference is correct. If the flexible interpretation is unsupported, mark the problem in Interpretation_Problem_Flag and lower Mapping (Rule_Mapping). Lower Citation (Evidence_Anchoring) only when the rationale misstates, over-specifies, fabricates, or materially distorts the script evidence itself.

Common quality adjustments and flags:

- If the script indicates that a symptom was present within the intended 1-2 week rating window, but the rationale treats it as entirely absent or irrelevant, lower Mapping (Rule_Mapping) and set the relevant problem flag, such as wrong_time_window, contradiction, or unsupported_score, depending on the source of the error.

- If a symptom had clearly resolved before the intended rating window, do not penalize the rationale for treating it as absent, provided that the rationale handles the timing accurately.

- If a resolved or past symptom is treated as currently active without acknowledging timing or resolution, lower Mapping (Rule_Mapping) and set Interpretation_Problem_Flag to wrong_time_window.

- Use of broader construct-level or cross-tag evidence is not itself an error. It becomes a problem only when the evidence is clinically irrelevant, temporally inappropriate, inconsistent with the target item, or used without acknowledging uncertainty where that uncertainty is important for the score.

- If the AI says "no mention" when a relevant utterance exists, lower Citation (Evidence_Anchoring) according to the degree of misrepresentation.

- If the AI says "clearly stated" when the script only supports a broader, adjacent, or inferred statement, lower Citation (Evidence_Anchoring) for over-specific anchoring and set Interpretation_Problem_Flag to overstatement.

\## Optional Descriptive Labels

Also assign these categorical labels. These are not scores but help interpret the scores.

- Evidence_Use_Label: none_or_absence_handled, generic_summary, close_paraphrase, direct_quote, fabricated_or_wrong

- Mapping_Problem_Flag: none, insufficient_dimensions, wrong_time_window, wrong_tag, contradiction, unsupported_score

- Interpretation_Problem_Flag: none, wrong_time_window, wrong_tag, cross_tag_overuse, overstatement, unsupported_expansion, contradiction

\## Data

\### Interview script

{{fullscripts}}

\### AI evaluation results

{{evaluation_result}}

The AI evaluation results are a JSON list or object containing, for each item:

- tag

- question

- evaluation_value

- evaluation_reason

\## Output Format

Return only valid JSON. Do not include markdown, code fences, comments, or explanatory text outside JSON.

Evaluate every tag in the AI evaluation results.

Use concise Korean for reason fields if the source script and AI rationale are in Korean.

Output schema:

Keep the JSON field names exactly as shown below. The display labels are Citation, Structure, Mapping, and Expansion.

[

{

"tag": "MDEA4-1-1",

"Evidence_Status": "",

"Citation": "",

"Citation_reason": "",

"Structure": "",

"Structure_reason": "",

"Mapping": "",

"Mapping_reason": "",

"Expansion": "",

"Expansion_reason": "",

"Evidence_Use_Label": "",

"Mapping_Problem_Flag": "",

"Interpretation_Problem_Flag": ""

}

]

```
