# Supplementary material for: Large Language Models for Distress Rating in Korean Psycho-Oncology Interviews: Exploratory Clinician-Benchmarked Evaluation Study
Source: J Med Internet Res. 2026 Sep 9;28:e91405. doi: 10.2196/91405 (PMC13557317; doi:10.2196/91405)
Supplement: Supplementary file 3 [file jmir-v28-e91405-s002.docx]

**Multimedia Appendix 2. De-identified examples of clinician–LLM disagreements adjudicated as ambiguous**

“Ambiguous” disagreements were defined as cases in which the transcript was inherently equivocal or did not provide sufficient information to determine confidently whether the LLM had made a definite binary classification error. The following three de-identified cases are presented as representative examples of ambiguous disagreements. The excerpts were translated from Korean, lightly condensed, and de-identified. Repetitions, filler expressions, and details unrelated to symptom severity were omitted, while hedging and frequency expressions relevant to scoring were retained.

**Case A. Perceived burden on family members**

**Target item:** If you felt that you were a burden to your family or others because of your cancer over the past 1-2 weeks, how severe was it?

**Translated and condensed interview excerpt**

Clinician: Do you feel that your cancer makes you a burden to your family or the people around you?
Patient: My children are doing well on their own, so that is not a major concern. However, because there are things I cannot manage on my own, I have to rely on [a family member], and that is what weighs on me the most. I do not want them to see me looking ill, but it shows in my expression. I feel most sorry about that. Other than that, not really.

**Ratings:** Clinician reference, 1; GPT-4o, 1; Claude 3.5, 2; Gemini 2.5, 2.

**Basis for ambiguous adjudication**

The patient described reliance on a family member as the aspect that weighed most heavily and repeatedly emphasized feeling sorry, indicating substantial emotional salience. However, the interview did not establish how frequently or how persistently this feeling occurred or whether it interfered with functioning. The strong salience of the concern could suggest greater-than-mild distress, whereas the absence of threshold-defining information and the statement “other than that, not really” supported a conservative score of 1.

**Case B. Fear of cancer progression or recurrence**

**Target item:** If you experienced fear of cancer recurrence or progression, how severe was it?

**Translated and condensed interview excerpt**

Clinician: Are you afraid that the cancer might progress or recur?
Patient: I have some fear.
Clinician: A little?
Patient: A little. Because it keeps spreading, and the numbers go down and then up and then things worsen again, I do wonder what will happen. I do have some concern about that.

**Ratings:** Clinician reference, 1; GPT-4o, 1; Claude 3.5, 2; Gemini 2.5, 2.

**Basis for ambiguous adjudication**

The patient clearly endorsed concern about progression in the context of repeated metastasis and fluctuating clinical values. However, the patient repeatedly qualified the fear as “some” or “a little,” and the transcript did not establish its frequency, duration, or functional impact. Thus, symptom presence was clear, but the available information did not adequately resolve whether its severity remained mild or crossed the threshold for a moderate rating.

**Case C. Palpitations, chest tightness, or shortness of breath**

**Target item:** If you experienced palpitations, chest tightness, or shortness of breath over the past 1–2 weeks, how severe was it?

**Translated and condensed interview excerpt**

Clinician: You have not had any palpitations, chest tightness, or shortness of breath?
Patient: But earlier, when I was lying down, I became short of breath. I do not know why.
Clinician: Was it very uncomfortable?
Patient: It was a little uncomfortable, so I sat up. Before coming here, I tried lying down for a while, but I became short of breath. It seems to happen when I am receiving [treatment].
Clinician: So it occurs during [treatment]?
Patient: I sometimes feel a little short of breath at home as well.
Clinician: At home as well?

**Ratings:** Clinician reference, 1; GPT-4o, 1; Claude 3.5, 2; Gemini 2.5, 1.

**Basis for ambiguous adjudication**

The patient described the symptom as occurring “sometimes” and being only “a little” uncomfortable, supporting a mild rating. However, the dyspnea occurred in more than one setting and was uncomfortable enough for the patient to sit up, providing a plausible cue of interference.
