# Supplementary material for: Large Language Models for Distress Rating in Korean Psycho-Oncology Interviews: Exploratory Clinician-Benchmarked Evaluation Study
Source: J Med Internet Res. 2026 Sep 9;28:e91405. doi: 10.2196/91405 (PMC13557317; doi:10.2196/91405)
Supplement: Supplementary file 4 [file jmir-v28-e91405-s003.docx]

**Multimedia Appendix 4. Supplementary Tables**

Table S1. Binary screening performance in the sensitivity analysis

|  |  | GPT | Claude | Gemini |
| --- | --- | --- | --- | --- |
| Binary | Sensitivity | 0.895 (0.861-0.925) | 0.911 (0.881-0.936) | 0.867 (0.829-0.899) |
|  | Specificity | 0.978 (0.971-0.984) | 0.957 (0.946-0.968) | 0.956 (0.944-0.966) |
|  | PPV | 0.902 (0.873-0.925) | 0.829 (0.787-0.867) | 0.817 (0.764-0.862) |
|  | NPV | 0.976 (0.969-0.983) | 0.979 (0.972-0.986) | 0.969 (0.958-0.978) |
|  | *F*_1_-score | 0.898 (0.874-0.919) | 0.868 (0.838-0.893) | 0.841 (0.802-0.872) |
|  | Adjusted *P* | .88 | .007 | .049 |

Values are estimates (95% CIs). The 170 patient-item pairs classified as ambiguous for at least one model were excluded identically from all three model datasets, leaving 3,761 ratings per model from 101 patients. Scores were dichotomized as 0-1 versus ≥2. Confidence intervals were calculated using nonparametric patient-level cluster bootstrapping with 2,000 resamples. NPV, negative predictive value; PPV, positive predictive value.

Table S2. Distributions of ambiguous mismatches by model and clinical domain

| Clinical domain | GPT-4o | Claude 3.5 | Gemini 2.5 | Total |
| --- | --- | --- | --- | --- |
| All domains | 97/248 (39.1%) | 88/301 (29.2%) | 118/355 (33.2%) | 303/904 (33.5%) |
| Psychiatric symptoms | 79/187 (42.2%) | 70/236 (29.7%) | 97/276 (35.1%) | 246/699 (35.2%) |
| Physical symptoms | 6/29 (20.7%) | 10/30 (33.3%) | 7/29 (24.1%) | 23/88 (26.1%) |
| Psychiatric interference | 9/20 (45.0%) | 5/19 (26.3%) | 7/24 (29.2%) | 21/63 (33.3%) |
| Physical interference | 3/12 (25.0%) | 3/16 (18.8%) | 7/26 (26.9%) | 13/54 (24.1%) |

Values are presented as the number of ambiguous mismatches/total adjudicated binary mismatches (%). Percentages represent the proportion classified as ambiguous among mismatches.

Table S3. Patient-clustered GEE tests of model and clinical-domain effects on ambiguous errors

| Effect | Wald χ² | df | *P* | Pairwise Comparison |
| --- | --- | --- | --- | --- |
| Model | 6.576 | 2 | .04 | GPT-4o vs Claude 3.5 (Adjusted *P*=.03)  GPT-4o vs Gemini 2.5 (Adjusted *P*=.24)  Gemini 2.5 vs Claude 3.5 (Adjusted *P*=.24) |
| Clinical domain | 2.381 | 3 | .50 | Not applicable |
| Model × clinical domain | 6.570 | 6 | .36 | Not applicable |

The outcome was whether an adjudicated binary mismatch was classified as ambiguous. Binomial logistic generalized estimating equations were fitted with patient-level clustering, an exchangeable working correlation structure, and robust standard errors. Model and clinical domain were included simultaneously in the additive model. The model-by-domain interaction was evaluated in a separate interaction model. Pairwise model contrasts were performed following the significant global model test and were adjusted using the Holm procedure.

Table S4. Patient characteristics and model-specific heterogeneity in ambiguous errors

| Patient Characteristic | Effect | Wald χ² | df | Adjusted *P* |
| --- | --- | --- | --- | --- |
| Age | Main effect | 0.002 | 1 | .97 |
| Age | Model × characteristic | 0.238 | 2 | >.99 |
| Clinician-rated item burden | Main effect | 2.596 | 1 | .21 |
| Clinician-rated item burden | Model × characteristic | 0.727 | 2 | >.99 |
| MDE status | Main effect | 3.513 | 1 | .18 |
| MDE status | Model × characteristic | 0.240 | 2 | >.99 |
| Sex, female vs male | Main effect | 5.422 | 1 | .08 |
| Sex, female vs male | Model × characteristic | 1.115 | 2 | >.99 |

Holm adjustment was applied separately to the four patient-characteristic main-effect tests and the four model-by-characteristic interaction tests. Age and clinician-rated item burden were standardized. MDE, major depressive episode.

Table S5. Cross-classified mixed-effects models examining associations between meta-evaluation scores and absolute error, with random intercepts for patients and distress items

|  | GPT |  | Claude |  | Gemini |  |
| --- | --- | --- | --- | --- | --- | --- |
|  | β | Adjusted *P* | β | Adjusted *P* | β | Adjusted *P* |
| Citation | 0.055 | <.001 | 0.028 | .01 | 0.013 | .24 |
| Structure | 0.084 | <.001 | 0.116 | <.001 | 0.117 | <.001 |
| Mapping | -0.157 | <.001 | -0.269 | <.001 | -0.210 | <.001 |
| Expansion | 0.090 | <.001 | 0.044 | .01 | 0.097 | <.001 |

β coefficients represent fixed-effect estimates from cross-classified mixed-effects models, reflecting the association between each meta-evaluation score and absolute error. Adjusted *P* values indicate false discovery rate-adjusted *P* values across meta-evaluation dimensions within each model.

Abbreviations: GPT, generative pre-trained transformer.
