# Supplementary material for: Large Language Models for Distress Rating in Korean Psycho-Oncology Interviews: Exploratory Clinician-Benchmarked Evaluation Study
Source: J Med Internet Res. 2026 Sep 9;28:e91405. doi: 10.2196/91405 (PMC13557317; doi:10.2196/91405)
Supplement: Supplementary file 5 [file jmir-v28-e91405-s004.docx]

**Multimedia Appendix 5. Supplementary Figures**

Figure S1. Ordinal confusion matrices comparing model-generated symptom severity ratings (0-3 scale) with clinician reference ratings

Cell values represent the frequency of exact matches and directional deviations, allowing visualization of agreement patterns and severity-level misclassification beyond binary thresholds.

Figure S2. Domain-adjusted mixed-effects estimates based on automated and human-reference meta-evaluation scores.

Panels A, B, and C present results for GPT-4o-, Claude 3.5-, and Gemini 2.5-generated rationales, respectively. Points represent fixed-effect coefficients (β), and horizontal lines indicate 95% confidence intervals. Black circles show estimates based on the mean scores independently assigned by GPT-5.4, Claude Sonnet 4.6, and Gemini Pro 3.1 in the full 101-patient cohort. Blue squares show corresponding estimates in the 10-patient subset, and orange triangles show estimates based on human-reference ratings in the same subset. All four meta-evaluation dimensions were entered simultaneously, with clinical domain included as a fixed effect and crossed random intercepts specified for patients and items. Positive coefficients indicate associations with larger absolute error, whereas negative coefficients indicate associations with smaller absolute error. Filled markers indicate Benjamini-Hochberg-adjusted *P*<.05, and open markers indicate *P*≥.05. The vertical line denotes β=0. GPT, generative pre-trained transformer.
